# Abnormal Growth and Feeding Behavior in Upper Airway Obstruction in Rats

**DOI:** 10.3389/fendo.2018.00298

**Published:** 2018-06-04

**Authors:** Ariel Tarasiuk, Yael Segev

**Affiliations:** ^1^Sleep-Wake Disorders Unit, Soroka University Medical Center, Beer-Sheva, Israel; ^2^Department of Physiology, Faculty of Health Sciences, Ben-Gurion University of the Negev, Beer-Sheva, Israel; ^3^Shraga Segal Department of Microbiology and Immunology, Ben-Gurion University of the Negev, Beer-Sheva, Israel

**Keywords:** sleep-disordered breathing, upper airway obstruction, sleep, growth, metabolism, rats

## Abstract

Pediatric obstructive sleep apnea (OSA) is a syndrome manifesting with snoring and increased respiratory effort due to increased upper airway resistance. In addition to cause the abnormal sleep, this syndrome has been shown to elicit either growth retardation or metabolic syndrome and obesity. Treating OSA by adenotonsillectomy is usually associated with increased risk for obesity, despite near complete restoration of breathing and sleep. However, the underlying mechanism linking upper airways obstruction (AO) to persistent change in food intake, metabolism, and growth remains unclear. Rodent models have examined the impact of intermittent hypoxia on metabolism. However, an additional defining feature of OSA that is not related to intermittent hypoxia is enhanced respiratory loading leading to increased respiratory effort and abnormal sleep. The focus of this mini review is on recent evidence indicating the persistent abnormalities in endocrine regulation of feeding and growth that are not fully restored by the chronic upper AO removal in rats. Here, we highlight important aspects related to abnormal regulation of metabolism that are not related to intermittent hypoxia *per se*, in an animal model that mimics many of the clinical features of pediatric OSA. Our evidence from the AO model indicates that obstruction removal may not be sufficient to prevent the post-removal tendency for abnormal growth.

## Introduction

Obstructive sleep-disordered breathing includes a spectrum of clinical entities with variable severity ranging from primary snoring to obstructive sleep apnea (OSA) (1, 2). Children with OSA suffer from upper airway obstruction (AO) during sleep that is manifested by increased respiratory efforts, large variations in intrathoracic pressure (up to −50 mmHg during peak inspiration), intermittent hypoxia, ultimately leading to sleep fragmentation and nonrestorative sleep. OSA is relatively common in children, and it may have serious consequences on longitudinal growth, body weight, energy metabolism, cardiovascular and neurobehavioral abnormalities, and increased health-care utilization (1–10). Estimates of OSA prevalence range between 1 and 5.7% depending mainly on the populations studied (1, 2, 11, 12). It is estimated that 5–56% of OSA cases develop growth retardation, with the lower prevalence probably reflecting increased awareness and earlier diagnosis and treatment (13–17). The mechanisms underlying the development of growth retardation in OSA continue to be highly controversial. Three main possibilities have been put forward to explain growth retardation in OSA. First, it is possible that dysphagia (18) is due to enlarged tonsils and adenoids, and decreased appetite due to changes in olfactory acuity in some cases. Second, it has been postulated that dysregulation of energy supply/energy expenditure balance (3, 18–20), due to the increased respiratory efforts (work of breathing) during sleep, will lead to increased metabolic expenditure and contribute to slow weight gain in these children (3). However, this mechanism has been disputed, as total energy expenditure was not affected by OSA (21). Third, more recently, impaired homeostasis of hormones such as growth hormone (GH), ghrelin, and leptin has been reported (4, 5, 22–25). The GH homeostasis is recognized as a key mechanism underlying impaired longitudinal growth (1, 4, 5). GH secretion occurs in pulses from the anterior pituitary somatotropic cells mainly during deep slow wave sleep onset (26, 27). Deep slow wave sleep is initiated in the preoptic area of the hypothalamus and consists of delta electroencephalogram activity, i.e., high-amplitude brain waves with a frequency of oscillation between 0.5 and 4 Hz (23, 28–31). OSA has also been shown to cause growth failure in some young children, and metabolic syndrome and obesity were reported in other cases (1, 3, 5, 6, 13, 15, 16, 18). OSA is most prevalent in 2- to 8-year-old children, when tonsil and adenoid volume is largest relative to the upper airway diameter; these children are usually referred to adenotonsillectomy as the first-line treatment (1, 3, 6, 15, 32). A currently poorly understood phenomenon is the fact that treatment of OSA can lead to accelerated weight gain in children, i.e., it normalizes weight in children who have failure to thrive, but increases the risk for obesity in overweight patients (1, 2, 5, 6, 15, 32–35). Regulation of energy expenditure is multifactorial and includes factors such as metabolic rate at rest, physical activity, and thermic effect of food intake (19, 20, 36, 37). Whole-body energy balance to promote weight gain may be altered following treatment of OSA (19, 20). However, study design and the between-group variability make a conclusion on the effect of treatment difficult. Although adenotonsillectomy in children and positive airway pressure (in adults) treatments predispose humans to a positive energy balance and accelerate body weight gain, sedentary lifestyles, dietary intake, and selection of high caloric/glycemic index foods may have greater impacts on weight change (6, 19, 20, 38–40). However, the majority of clinical studies concentrated on elucidating the endocrine consequences of the surgical treatment while data on normal healthy controls barely exist.

Experimental models of sleep apnea provide mechanistic insight into the apnea generation as well as into its impact on cardiovascular, metabolic, and psychological consequences (41, 42). The commonly used model to study OSA involves implementation of intermittent hypoxia, i.e., the repetitive brief hypoxic episodes like those that occur in OSA (43) or specifically dusting sleep (44, 45) to explore the impact of intermittent hypoxia on cardiovascular (43, 46), sleep (47, 48), and neurocognitive parameters (42). Another defining feature of OSA, however, is the mechanical changes in work of breathing that are not associated with intermittent hypoxia. To elucidate the role of the mechanical load, we first developed a model of mechanical obstruction in rodents in 1991 (49). We used this model 2 years later to elucidate the impact of chronic upper AO on cardiac function in rats (50). Here, we highlight important aspects related to abnormal regulation of metabolism that are not related to intermittent hypoxia *per se*, in an animal model that features many of the clinical signs of pediatric OSA.

## The Upper AO Model

In the AO model, respiratory load is surgically induced in 22-day-old rats by tracheal narrowing and animals were followed up to 7 weeks; this period is comparable to half a year up to 20 years in humans (25). AO induces adaptive response in the respiratory system including alterations in respiratory muscles (49, 51), control of ventilation (52, 53), sleep, growth, and metabolism (23–25, 31, 54–57). Following surgery trachea diameter was reduced by 45%, its resistance increased by 46–100%, and respiratory effort more than doubled (23, 52, 53, 55); following obstruction removal, trachea diameter was normalized to control values (25). One of the limitations of this model is the fact that respiratory loading was both inspiratory and expiratory and not sleep related, resembling pediatric subglottic stenosis. In clinical OSA, however, the upper AO is predominately inspiratory and manifested mainly during sleep (58). The similarity of this model and sleep apnea are striking as obstructed animals exhibit sleep fragmentation and abnormal growth similar to OSA (1, 2, 23, 54, 55). The AO elicits audible wheezing, especially following activity, whereas no sign of respiratory distress was observed at rest (49, 50, 53). Under these conditions, animals maintain PO_2_ in the normal range with no evidence for gas exchange abnormalities (24, 25, 50, 52, 54, 57), hemoglobin and lactate levels (23, 25, 52, 54, 57), and daily food intake were higher (25, 53, 54). By contrast, intermittent hypoxia can lead to decreased food intake, erythropoiesis, and liver injury (59–65). In children with OSA, oxygen saturation may decrease with airway loading during sleep (66). However, in AO, no changes were found in liver enzymes, liver histology, prolyl-hydroxylase 2, or hypoxia-inducible factors (25). These findings support the notion that intermittent hypoxia does not play a role in this abnormal energy metabolism and growth observed in the AO animal model.

## Orexin and Breathing

**Figure 1:**
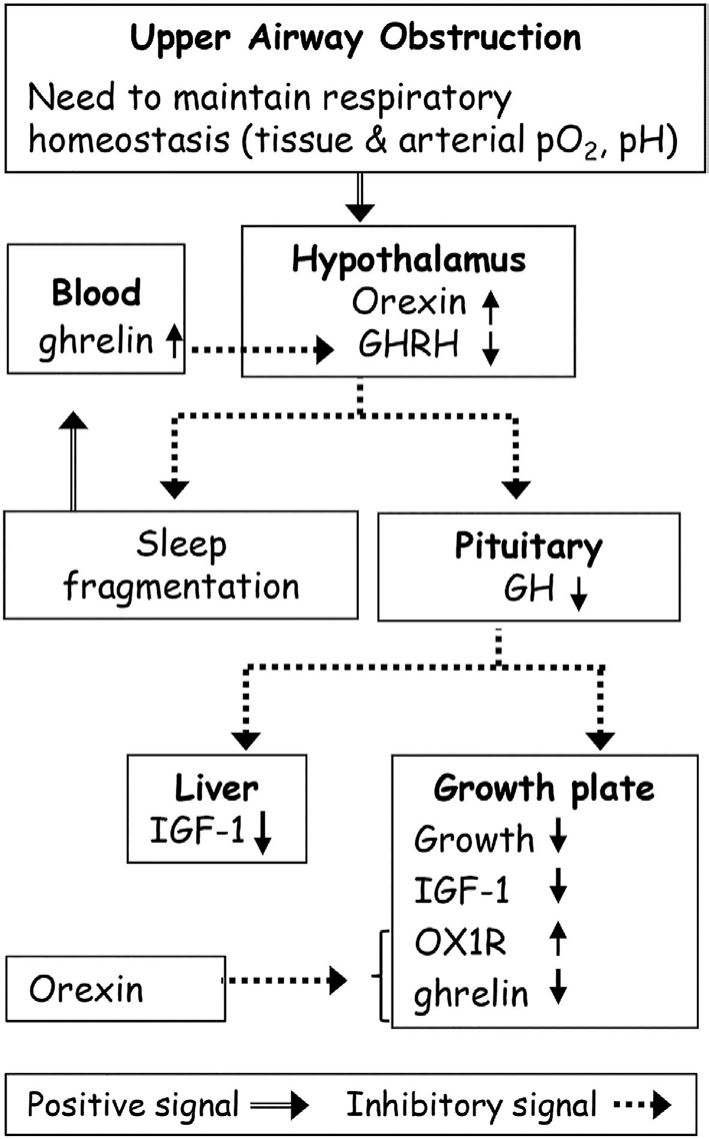
Possible effects of upper airway obstruction (AO) on sleep and growth. Upper AO leads to adaptive changes in the respiratory mechanics to maintain respiratory homeostasis. Orexin plays a role in maintaining breathing homeostasis in AO *via* its primary role in carbon dioxide chemoreception. Orexin inhibits growth hormone (GH) release from the pituitary gland through inhibition of hypothalamic hormone-releasing hormone (GHRH) neurons. Abnormalities in GHRH underlie both growth and sleep disorders in AO. The continuous ghrelin elevation in AO possibly due to partial sleep loss and increased orexin was sufficient to desensitize the hypothalamic–pituitary–GH axis. AO causes suppression of the GH axis, and of the global and local growth plate IGF-1 levels leading to growth retardation. Orexin receptor 1 (OX1R) plays a role in growth retardation by modulation of local ghrelin levels.

Airways obstruction leads to adaptive changes in the respiratory mechanics including large inspiratory swings in pleural pressure and increased diaphragmatic contractile force (49, 50) to maintain respiratory homeostasis (Figure 1) (25, 52–54). These adaptations are critical especially during sleep, a condition where respiratory muscle force may not be sufficient to support obstructed ventilation. AO leads to increased hypothalamic orexin level, wakefulness, and hunger (23–25). The orexins (orexin A and orexin B) are novel hypothalamic peptides derived from the common precursor prepro-orexin that acts through two subtypes of receptors [orexin receptor 1 (OX1R) and OX2R]. Orexin neurons have emerged as a key orchestrator of sleep–wake activity, and breathing and feeding centers receive stimulatory inputs from orexin neurons (67–70). Orexin neurons are active during wakefulness but show little or no activity during paradoxical sleep (rapid eye movement sleep in humans) and slow wave sleep (70–72). Orexin-containing neurons located in the lateral hypothalamus contribute to carbon dioxide (CO_2_) chemoreception (73–77) in a vigilance-state- and diurnal-cycle-dependent manner (73, 76). Prepro-orexin knockout mice exhibit a large decrease in the ventilatory response CO_2_ during wakefulness that contributes to a higher arterial PCO_2_ (78). Hypothalamic orexin cell firing can be stimulated by extracellular pH levels, at least partial closure of TASK-like channels (74), and possibly *via* acid-sensing ion channels (77, 79). Respiratory acidosis produced by OSA causes arousal and stimulates breathing, which normalizes extracellular levels of protons (79). In the AO model, increase in hypothalamic orexin plays an important role in maintaining respiration (23, 24, 54) and increased food intake (24, 25). Administration of almorexant (dual orexin receptor antagonist) normalized sleep (54) but induced severe breathing difficulties in the AO group, while it affected neither sleep nor breathing in control animals. Similar breathing difficulties were found during recovery sleep from 4-h sleep deprivation protocol when sleep was stimulated and orexin level is minimal. Orexin plays a role in maintaining breathing homeostasis in AO, probably *via* its primary role in CO_2_ chemoreception (76). Further studies are needed to quantify the role of TASK-like channels (74) and acid-sensing ion channels in regulation of breathing in AO model. Leptin has an important role in central chemoreception; leptin deficiency in ob/ob mice produces marked depression of the hypercapnic ventilatory response (62). In AO rats, however, leptin decreases and does not play a role in respiratory regulation (25, 54).

## Orexin–GH Axis and Feeding

The need to maintain ventilation in AO is associated with upregulation of hypothalamic orexin, which causes suppression of the growth hormone-releasing hormone/growth hormone axis (GHRH/GH) and decreased sleep (Figure 1) (23, 31, 54). AO disruption of sleep leads to adverse health outcomes in rodents, i.e., increased appetite hormones (orexin, ghrelin) and food intake and GH suppression (23–25). Orexin serves as an important link between peripheral metabolism and homeostatic challenges including sleep, respiration, feeding, and neuroendocrine homeostasis (67–70, 80–82). Orexin-A inhibits GH release from the pituitary gland through inhibition of GHRHergic neurons of the periventricular nucleus and of the arcuate nucleus and stimulation of somatostatin in the hypothalamus (82–85). Enhanced GH secretion and induction of sleep are two parallel and closely interrelated consequences of hypothalamic GHRHergic neurons activation (29, 86, 87). Abnormalities in GHRH levels could underlie both growth and sleep disorders in AO (23). The decreased GHRH content in AO was related to increased hypothalamic orexin and somatostatin levels, and in obstruction removal group to increased somatostatin (25, 54). Administration of ritanserin (selective 5HT2 antagonist) normalized both GHRH content and slow wave sleep (23, 31, 54).

Interestingly, AO elicits sustained ghrelin elevation, sufficient to desensitize the hypothalamic–pituitary–GH axis while still causing hyperphagia (25). This hyperphagia was also attributed to upregulation of orexin and mediators that are activated by ghrelin, such as neuropeptide Y and agouti-related peptide, and to decreased circulatory leptin levels. Gut-derived ghrelin both stimulates feeding behavior and causes release of GHRH from the hypothalamus in response to fasting (88–90) by activation of the GH-secretagogue receptors (91). At least three types of neurons, such as GHRHergic, neuropeptide Y, and agouti-related peptidergic, are well-defined targets for ghrelin action on feeding (92). Acceleration of growth by ghrelin depends on its level and pattern of secretion (93). Short exposure to ghrelin causes augmentation of GH release and increases appetite *via* the hypothalamic GHRH receptor, while continuous exposure increases feeding but suppresses the GH release. The most prevalent explanation of the impaired somatic growth in OSA patients is that their GH homeostasis is disturbed (4). A meta-analysis study (5) found the evidence of improved endocrine homeostasis of the GH axis that is associated with improved somatic growth following adenotonsillectomy, supporting the concept that GH homeostasis is impaired in pediatric OSA. However, the limitation of these studies was that the comparison has been done to hormone levels in the short time windows before and after the surgical intervention, not including healthy controls. Levels of IGF-1 and of its main binding protein, IGF-binding protein 3, are well-established predictors of the mean GH levels across the day (94–96). One of the interesting findings in the AO model is that following the obstruction removal, the GH/IGF-1 axis does not normalize and these animals still exhibit substantial growth retardation (25). Both GHRH and IGF-1 levels do not reach control values and obstruction removal animals continue to exhibit shorter body length despite normalized tracheal diameter, indicating persistent deregulation of GH axis (25). Increased food intake in AO was probably a physiological adaptation required to provide the extra energy needed for increased additional wakefulness (23, 31, 36, 54) and possibly elevated energy expenditure due to increased work of breathing (49, 51). Increased feeding behavior was associated with persistent remodeling of the appetite homeostasis long after the successful removal of the upper AO (25). It is possible that AO (treated or untreated) leads to persistent alterations of appetite homeostasis and increases the preference for a carbohydrate-rich diet. Further studies are needed to explore the whole-body energy balance using an open-circuit indirect calorimeter (36) and to find out whether AO obstruction removal affects nutritional preference for carbohydrate-rich vs. high-fat diet that in turn may contribute to abnormal body weight gain. In children, despite resuming normal sleep and ventilation by adenotonsillectomy, the risk for obesity remains high (3, 6, 32–35). This has been attributed to a shift toward less active lifestyle and, possibly, to unhealthy food choices, highlighting the importance of changing the lifestyle following surgery (6, 19, 32, 38–40).

## Metabolic Consequences of AO

Airways obstruction leads to partial sleep loss, i.e., 30–45% elevation of wake duration, from early life to adulthood (23, 24, 31, 54). Low weight gain and growth were observed in AO animals despite increased energy intake and increased intestine surface area to absorb water and nutrients (25, 54), similar to the effect observed during chronic partial sleep loss in rats (97). Sleep fragmentation, regardless of the methodology, including the AO in rats, leads to considerable pathophysiological abnormalities including decreased body temperature (98), decreased GHRH/GH axis (27, 99), and increased feeding behavior (61, 97). Similar sleep abnormalities in humans may increase risk for type 2 diabetes, obesity, and cardiovascular diseases (100, 101). The difference in metabolic response to sleep abnormalities between humans and rats may simply reflect an inter-species difference (102), although other contributing factors such as intermittent hypoxia cannot be excluded in the case of sleep apnea (1, 2, 22). Moreover, the different levels of physical activity in human (6) and AO animals may also play a role. A striking reduction of adipose tissue content and distribution was the main factor contributing to the slow body weight gain in AO rats (54). Slower weight gain (or even weight loss) was also reported in earlier studies of sleep deprivation in rodents, but these reports were not consistent. Thus, whereas most studies showed an increase in food intake (97, 103), some groups reported no change in energy intake following sleep loss (102). Short sleep duration augments gut-derived ghrelin and appetite (99). Ghrelin drives motivated behavior such as motivated movement, feeding, and increased arousal (88, 104). The essential need to provide the energy required to support loaded breathing in AO (49) accompanied by enhanced wakefulness (36) makes the increased feeding essential for physiological adaptation to the chronic respiratory condition.

## Bone Metabolism and Architecture

Growth retardation is highly prevalent in children suffering from OSA. It aging adults, OSA has been shown to affect bone metabolism and bone mass *via* decreased sleep quality, nocturnal hypoxia, inflammation, etc. (105). The endocrine effect of GHRH/GH is induction of IGF-1 in various organs, including the epiphyseal growth plate (EGP) in bones (106). AO causes a significant suppression of the GH axis, and of the global and local EGP IGF-1 levels (Figure 1) (55, 57). The global IGF-1 levels are primarily determined by liver synthesis; other peripheral tissues contribute somewhat to circulating global IGF-1 levels but their contributions are less significant (107). Local IGF-1 mediates the GH effect on the EGP by binding to the specific receptors (94, 108). Reduced local IGF-1 release in the EGP will lead to growth retardation (109).

We assessed somatic growth by measuring long bones length and by microCT scanning (24, 55, 110). Longitudinal growth is a well-orchestrated process that occurs *via* endochondral ossification at the growth plates (111). In AO animals, growth gain was inversely correlated with enhanced upper airways resistance (55). Both proliferative and hypertrophic zones were narrowed in the AO animals and these animals exhibited reduced bone mass. These pathophysiological bone abnormalities were related to EGP failure and dysregulation of the levels of endochondral ossification markers (e.g., collagen type II, X, Sox9, and RankL) (24). Pharmacological stimulation of GH release restored local EGP IGF-1. The growth parameters were only partially restored (55), however, indicating that other pathways are involved. Seeking the identity of these pathways, we focused our attention on orexin and ghrelin peptides in AO animals. OX1R has been shown to play an important role in bone remodeling and metabolism by modulation of local ghrelin levels (112). Several ghrelin related signaling pathways, including phosphatidylinositol 3-kinase/AKT, apparently are involved in bone metabolism and development (113, 114). Orexins activate transcription factor peroxisome proliferator-activated receptor gamma (PPARγ) in bone marrow adipose tissue, causing bone mass loss and leading to fatty bone marrow (115–117). Obstruction removal and administration of almorexant normalized sleep but did not reverse the phenomena (24); these finding indicate persistent bone remodeling that is not related to loss of developmental time *per se*. Increased expression of orexin and OX1R, and suppression of GH-secretagogue receptor and local ghrelin in the EGP are the main mechanisms contributing to increased adipocyte differentiation and fatty bone marrow found in AO. Dual orexin receptor antagonist considerably improved EGP growth and restored sleep in AO animals. Our findings support the possibility that OX1R plays a significant role in bone development in AO animals (Figure 1) (24). The robust increase of PPARγ and reduction in Sox9 levels strongly suggest that the course of early bone development has been impaired (118). Orexins have been shown capable to stimulate marrow PPARγ that in return upregulates marrow adipogenesis and causes bone mass loss (112, 115, 116). Downregulation of Sox9 also promotes adipocyte differentiation, a process that is activated by PPARγ (119).

## Conclusion

The AO model provides mechanistic insight about functional interactions between orexin and the hypothalamic–pituitary axis in regulation of sleep, breathing, and growth. AO leads to adaptive changes in the respiratory mechanics to maintain respiratory homeostasis. The available information supports the notion that AO leads to increased hypothalamic orexin release, which plays an important role in maintaining respiration, but elicits sleep, energy metabolism, and growth abnormalities.

## Author Contributions

AT and YS wrote the manuscript and recruited funds.

## Conflict of Interest Statement

The authors have declared that no conflict of interest exists and none of the material in this manuscript has previously been published. The reviewer JJ and handling Editor declared their shared affiliation.
